# Properties of the Permeability Transition of Pea Stem Mitochondria

**DOI:** 10.3389/fphys.2018.01626

**Published:** 2018-11-21

**Authors:** Valentina De Col, Elisa Petrussa, Valentino Casolo, Enrico Braidot, Giovanna Lippe, Antonio Filippi, Carlo Peresson, Sonia Patui, Alberto Bertolini, Valentina Giorgio, Vanessa Checchetto, Angelo Vianello, Paolo Bernardi, Marco Zancani

**Affiliations:** ^1^Department of Agricultural, Food, Environmental and Animal Sciences (Di4A), University of Udine, Udine, Italy; ^2^Department of Biomedical Sciences, University of Padova and CNR Neuroscience Institute, Padova, Italy; ^3^Department of Biology, University of Padova, Padova, Italy

**Keywords:** Ca^2+^, cyclophilin, cyclosporin A, F-ATP synthase, permeability transition, plant mitochondria

## Abstract

In striking analogy with *Saccharomyces cerevisiae*, etiolated pea stem mitochondria did not show appreciable Ca^2+^ uptake. Only treatment with the ionophore ETH129 (which allows electrophoretic Ca^2+^ equilibration) caused Ca^2+^ uptake followed by increased inner membrane permeability, membrane depolarization and Ca^2+^ release. Like the permeability transition (PT) of mammals, yeast and Drosophila, the PT of pea stem mitochondria was stimulated by diamide and phenylarsine oxide and inhibited by Mg-ADP and Mg-ATP, suggesting a common underlying mechanism; yet, the plant PT also displayed distinctive features: (i) as in mammals it was desensitized by cyclosporin A, which does not affect the PT of yeast and Drosophila; (ii) similarly to *S. cerevisiae* and Drosophila it was inhibited by Pi, which stimulates the PT of mammals; (iii) like in mammals and Drosophila it was sensitized by benzodiazepine 423, which is ineffective in *S. cerevisiae*; (iv) like what observed in Drosophila it did not mediate swelling and cytochrome *c* release, which is instead seen in mammals and *S. cerevisiae*. We find that cyclophilin D, the mitochondrial receptor for cyclosporin A, is present in pea stem mitochondria. These results indicate that the plant PT has unique features and suggest that, as in Drosophila, it may provide pea stem mitochondria with a Ca^2+^ release channel.

## Introduction

According to Mitchell (1961), the bulk synthesis of ATP in mitochondria occurs by a chemiosmotic coupling of substrate oxidation with ADP phosphorylation. This ability is based on the highly selective and regulated permeability of the inner membrane. Oxidation of reduced substrates and electron transfer through the electron transport chain allows the generation of a proton motive force, which is then utilized by the mitochondrial F-ATP synthase to synthetize ATP. Before the emergence of chemiosmotic concepts, it had been reported that mitochondria could undergo a sudden permeability increase of the inner membrane, resulting in mitochondrial swelling (Raaflaub, 1953a,b). This feature has been further characterized in several studies and in the late 1970s Haworth and Hunter (Haworth and Hunter, 1979; Hunter and Haworth, 1979a,b) named it Permeability Transition (PT) and proposed that it was mediated by a channel, the Permeability Transition Pore (PTP). However, the general acceptance of the chemiosmotic theory led to the widespread view that the presence of a mechanism that would disrupt the proton gradient and prevent the synthesis of ATP could be an *in vitro* artifact. This view changed in the late 1980s when cyclosporin A (CsA) was discovered as an inhibitor of PT (Fournier et al., 1987; Crompton et al., 1988; Broekemeier et al., 1989; Davidson and Halestrap, 1990). In recent years the PT has gained increasing attention due to its association with several conditions and diseases in mammals, e.g., reperfusion heart injury and muscular dystrophies (Bernardi, 2013). In mammalian mitochondria opening of the PTP requires matrix Ca^2+^ and is favored by matrix Pi, thiol oxidants, cyclophilin D (CyPD), benzodiazepine 423 (Bz-423), while it is inhibited by Mg^2+^, thiol reductants, ADP and ATP (Bernardi et al., 2015).

In plants, a collapse of transmembrane electrical potential (ΔΨ), induced by Ca^2+^ and delayed by CsA, has been described in isolated pea stem mitochondria (Vianello et al., 1995). Subsequently, PT was observed in several plants, although it showed different features, depending on tissues and species. In potato tuber mitochondria Fortes et al. (2001) showed a Ca^2+^-induced PT that is CsA-insensitive, while Arpagaus et al. (2002) reported that PT is induced by Ca^2+^ and Pi and is inhibited by CsA, possibly through interaction with CyPD. In mitochondria from oat leaves (Curtis and Wolpert, 2002) and wheat roots (Virolainen et al., 2002), PT appears as a CsA-insensitive ΔΨ collapse accompanied by matrix swelling induced by Ca^2+^ and Pi. Mitochondrial Ca^2+^ uptake requires the addition of the Ca^2+^/H^+^ ionophore A_23187_ in oat, while it occurs spontaneously in wheat. Finally, Ca^2+^-dependent PTP opening has been shown to play a fundamental role in salt stress response in Arabidopsis *in situ* (Zhao et al., 2013). As such, plants show a diverse PTP phenomenology, but fundamental features are shared with those observed in animals. Among them, one of the most remarkable consequences (related to matrix swelling) is the release of cytochrome *c* (Cyt *c*) in the cytosol, leading to the onset of programmed cell death, shared as a common characteristic between yeast, insects, mammals, and plants (Balk et al., 1999; Robertson et al., 2002; Arama et al., 2006; Giannattasio et al., 2008).

Permeability transition is a complex phenomenon, and the responsible mechanism remains under debate. An intriguing hypothesis, which is independent of the molecular nature of the PTP, has suggested the PTP to be the result of a molecular exaptation in evolution (Vianello et al., 2012). The physical components of PTP that were tentatively proposed in the past are the voltage-dependent anion channel (VDAC), the benzodiazepine receptor, the adenine nucleotide translocase (ANT) and the phosphate carrier. However, isolated mitochondria from different organisms, in which the expression of each of these proteins was suppressed, still showed PT (Kokoszka et al., 2004; Krauskopf et al., 2006; Baines et al., 2007; Gutiérrez-Aguilar et al., 2014; Šileikytė et al., 2014; Kamei et al., 2018), indicating that they are not essential for the PT to occur. Recent evidence shows that, in addition to their enzymatic and structural roles, F-ATP synthase may be involved in PTP formation in mammalian (Giorgio et al., 2013; Alavian et al., 2014), *Saccharomyces cerevisiae* (Carraro et al., 2014; Kamei et al., 2018) and *Drosophila melanogaster* (von Stockum et al., 2015) mitochondria. Whether a similar involvement of F-ATP synthase occurs in plants remains to be tested (Zancani et al., 2015).

This work was undertaken to examine the functional features of the PT in pea stem mitochondria in the light of recent advances in the field, and to draw systematic comparisons with the PT in animals and yeast. The data suggest that this plant PTP shares several characteristics with those observed in other species yet has some unique features that allow some reflections on the evolution of PTP.

## Materials and Methods

### Plant Material

Etiolated pea (*Pisum sativum* L. cv. Meraviglia d’Italia, Ingegnoli) stems were obtained by growing seedlings on sand for 7 days, in the dark, at 25°C and 60% relative humidity.

### Isolation of Mitochondria

Crude mitochondria (CM) from pea stem were isolated from approximately 90 g FW of stems, homogenized in a mortar at 4°C in 120 plus 100 mL of extraction buffer (0.3 M sucrose, 20 mM HEPES-Tris [pH 7.6], 1 mM EGTA, 1 mM DTE, 0.6% [w/v] PVPP, 0.1% [w/v] BSA). The homogenate was filtered through six layers of cellulose gauzes and centrifuged at 2,500 *g* (SS34 Sorvall rotor) for 4 min at 4°C; the supernatant was then re-centrifuged at 28,000 *g* for 5 min. The pellet was re-suspended and homogenized in 120 mL of the extraction buffer, without PVPP, and again centrifuged at 2,500 *g* for 4 min. The supernatant was finally centrifuged at 28,000 *g* for 5 min. The resulting pellet was re-suspended in 1 mL (final volume) of 0.25 M sucrose, 10 mM MOPS-Tris (pH 7.4), 10 μM EGTA-Tris and 0.1% (w/v) defatted BSA (resuspension buffer). This fraction was kept on ice and immediately used in the following mitochondrial transmembrane electrical potential (ΔΨ), swelling, oxygen consumption, and Ca^2+^ retention capacity (CRC) experiments. For BN-PAGE, in-gel activity assay, SDS-PAGE and immunoblotting experiments mitochondria were further purified using Percoll gradients. CM from pea stem were isolated as described above, with minor changes, in extraction buffer (0.3 M sucrose, 20 mM HEPES-Tris [pH 7.6], 5 mM EGTA, 1 mM DTE, 1 mM PMSF in 1% [v/v] EtOH, 0.6% [w/v] PVPP, and 0.3% [w/v] BSA). CM were layered over a discontinuous Percoll density gradient (45, 21, and 13.5% [v/v]) and centrifuged at 20,000 *g* (HB-4 Sorvall rotor) for 40 min at 4°C. Purified mitochondria were collected at the 21–45% interface, washed three times in 150 mL of wash buffer (0.25 M sucrose, 10 mM Tris-HCl [pH 7.2]) and centrifuged at 28,000 *g* for 5 min. The pellet from Percoll-purified mitochondria (PM) was finally resuspended in 1 mL of wash buffer. Mitochondria were then frozen in liquid nitrogen and stored at -20°C.

### Isolation of Sub-Mitochondrial Particles

Sub-mitochondrial particles (SMP) were isolated by sonicating CM (1 mg protein ml^-1^) three times on ice-bath at 100 W for 30 s each pulse (Braun Labsonic 1510). The suspension was centrifuged at 28,000 *g* (SS34 Sorvall rotor) for 5 min and the supernatant was centrifuged at 120,000 *g* (Ty 70 Ti Beckman rotor) for 50 min. The final pellet was re-suspended in a buffer containing 0.25 M sucrose 10 mM MOPS-Tris (pH 7.4) and 0.1% (w/v) BSA.

### Membrane Potential Measurement

ΔΨ changes of CM were estimated by fluorescence quenching of safranine O or rhodamine 123 in a PerkinElmer LS50B spectrofluorimeter equipped with magnetic stirring, as described by Casolo et al. (2005). One mg of CM was incubated in 2 ml of resuspension buffer without BSA, containing 5 μM safranine O (or 0.15 μM rhodamine 123) and 10 μM of the Ca^2+^ ionophore ETH129 and incubated at 25°C. The wavelengths were set to 495 and 586 nm (2.5 nm slit width) for excitation and emission, respectively.

### Ca^2+^ Retention Capacity Detection

Ca^2+^ retention capacity (CRC) of CM was evaluated with the non-permeant fluorescent probe calcium green 5N (Fontaine et al., 1998). Addition of Ca^2+^ induces a fluorescence increase and mitochondrial Ca^2+^ uptake, which is followed as fluorescence decrease. Mitochondrial Ca^2+^ fluxes in CM were evaluated in a PerkinElmer LS50B spectrofluorimeter, equipped with magnetic stirring, incubating mitochondria under the same conditions as for ΔΨ measurements, but in the presence of 0.5 μM calcium green 5N (Molecular Probes), instead of safranine O, and incubated at 25°C. The excitation and emission wavelengths were set to 506 and 532 nm (2.5 nm slit width), respectively.

### Measurement of Mitochondrial Swelling

Swelling in CM was monitored as absorbance changes at 540 nm, following the method described by Pastore et al. (1999) and using an Agilent 8453 spectrophotometer. Mitochondria were re-suspended in the assay buffer (0.25 M sucrose or 0.125 M ionic osmoticum (NaCl, KCl, Choline-Cl), 10 mM MOPS-Tris [pH 7.4] and 10 μM EGTA-Tris) and incubated at 25°C.

### Assay of Proton Pumping and ATP Hydrolysis Activities

ATP-dependent proton transport in SMP was monitored as acridine orange (AO) fluorescence quenching. When the proton gradient is generated by ATPase proton-pumping activity in SMP, the fluorescent probe permeates the membranes and accumulates into the vesicles (Vianello et al., 1991). Fluorescence change was detected by a PerkinElmer LS50B spectrofluorimeter. The SMP (50 μg ml^-1^) were re-suspended in 2 mL of 0.25 M sucrose, 10 mM MOPS-Tris (pH 7.4), 50 mM KCl, 5 μM AO and incubated at 25°C. Divalent cations were added where indicated as MgCl_2_, MnCl_2_, and CaCl_2_. The reactions were started by the addition of 1 mM Tris-ATP. Hydrolysis of ATP in SMP was evaluated as Pi release following the method described by Cross et al. (1978), in the same buffer used for mitochondrial swelling measurements.

### Measurement of Mitochondrial Respiration

Oxygen consumption in CM was evaluated by a Clark-type oxygen electrode (YSI, Yellow Springs, OH, United States) as described in Casolo et al. (2005). One mg of CM was incubated in 2 mL of 0.25 M sucrose, 10 mM MOPS-Tris (pH 7.4), 10 μM EGTA-Tris, 1 mM Pi-Tris and 10 μM ETH129 and incubated at 25°C.

### Detection of Cytochrome *c* Release

Crude mitochondria were initially subjected to induction of PTP opening in 0.25 M sucrose or 0.125 M ionic osmoticum (NaCl, KCl, Choline-Cl), 10 mM MOPS-Tris (pH 7.4), 10 μM EGTA-Tris, 1 mM Pi-Tris and then the incubation mixture was collected from the cuvette and immediately used for the extraction of proteins at different timepoints. Samples were ultra-centrifuged at 100,000 *g* for 40 min by a Beckman L7-55 centrifuge (Ty 70 Ti rotor) and the mitochondria in the pellet were re-suspended in 50 mM Tris-HCl (pH 6.8). The soluble fractions in the supernatants were concentrated by 5000 MWCO VIVASPIN 6 (Sartorius) at 10,000 *g* (SM24 Sorvall rotor) for 40 min. Thirty micrograms of the concentrated proteins were later separated by 15% (w/v) SDS–PAGE and electroblotted onto a nitrocellulose membrane to detect the presence of the Cyt *c*. Blots were incubated with anti-Cyt *c* rabbit polyclonal antibody (1:1,000 dilution, from Agrisera). The reactive proteins were finally detected by nitroblue tetrazolium and 5-bromo-4-chloro-3-indolyl phosphate staining, after incubation with alkaline phosphatase-conjugated anti-rabbit IgG antibody (1:2,500 dilution, from Sigma).

### Blue Native (BN)-PAGE and In-Gel Activity Assay

Blue native-PAGE was performed according to Wittig et al. (2006). PM were solubilized by digitonin with a protein/detergent ratio of 1/2 that optimized the resolution of F-ATP synthase dimers in BN-PAGE. Native-PAGE precast gel (3–12% Bis-Tris protein gel) and buffers were purchased from Invitrogen (Thermo Fisher Scientific). The electrophoretic run was performed at 100 V with dark blue cathode buffer, replaced after 60–90 min by light blue cathode buffer, and stopped after further 6 h. In order to visualize the bands corresponding to the native F-ATP synthase forms, BN-PAGE gel was transferred into the zymogram buffer (35 mM Tris [pH 8.3], 270 mM glycine, 14 mM MgSO_4_, 8 mM Tris-ATP, 0.2% [w/v] Pb(NO_3_)_2_) and incubated overnight at 25°C.

### SDS-PAGE and Immunoblotting

Bands corresponding to the dimeric form of F-ATP synthase, identified by the zymogram, were excised from the BN-PAGE gel, subjected to 12% (w/v) SDS-PAGE and electroblotted onto nitrocellulose membranes. The following primary antibodies were used: anti-OSCP (polyclonal, 1:2000 dilution, from Santa Cruz Biotechnology), anti-CyP-D (monoclonal, 1:2,000 dilution, from Calbiochem). The secondary antibodies (1:10,000 dilution, from Sigma) conjugated to either horseradish peroxidase or alkaline phosphatase were used for enhanced chemiluminescence or colorimetric development, respectively.

### Protein Quantification

Protein concentration was estimated by colorimetric reaction with Bradford reagent (Bradford, 1976). The calibration curve was obtained using BSA as a standard.

### Reagents and Statistics

All chemicals were of the highest commercially available purity and, unless otherwise specified, purchased from Sigma-Aldrich. Results are typical of at least three independent replicates for each experiment, traces are representative of at least three independent experiments and bars in figures refer to the standard deviation. Statistical differences between each control and each treatment were performed by paired sample *t*-test, symbols ^∗^ and ^∗∗^ indicate values of *p* ≤ 0.05 and *p* ≤ 0.01, respectively. All statistical analyses were performed using the Statistica software ver. 10 (Statsoft Inc., Tulsa, OK, United States).

## Results

### Properties of the Permeability Transition in Pea Stem Mitochondria

**FIGURE 1:**
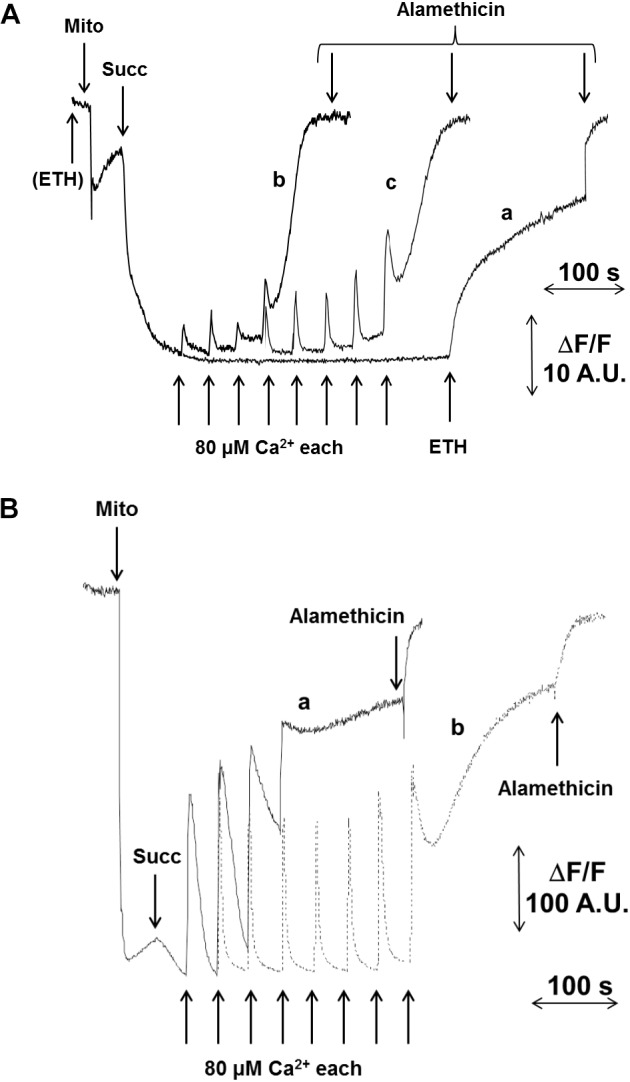
Effect of Ca^2+^ and CsA on PTP opening in CM. Isolated CM were incubated in 0.25 M sucrose, 10 mM MOPS-Tris (pH 7.4), 10 μM EGTA-Tris and 1 mM Pi-Tris. **(A)** The incubation medium was supplemented with 5 μM safranine O and ΔΨ was monitored as fluorescence decrease; in traces a, CaCl_2_ additions were done in the absence of ETH129; in traces b and c, 10 μM ETH129 was added to the incubation medium; in trace c, CsA was added at the concentration of 0.5 μM. **(B)** CRC was evaluated with 0.5 μM calcium green-5N as fluorescence increase in the presence of 10 μM ETH129. Where indicated, 5 mM succinate-Tris (Succ) and 80 μM Ca^2+^ pulses in sequence were added; in trace b, CsA was added at the concentration of 0.5 μM. In all traces, 5 μM alamethicin was added for the complete collapse of ΔΨ.

**FIGURE 2:**
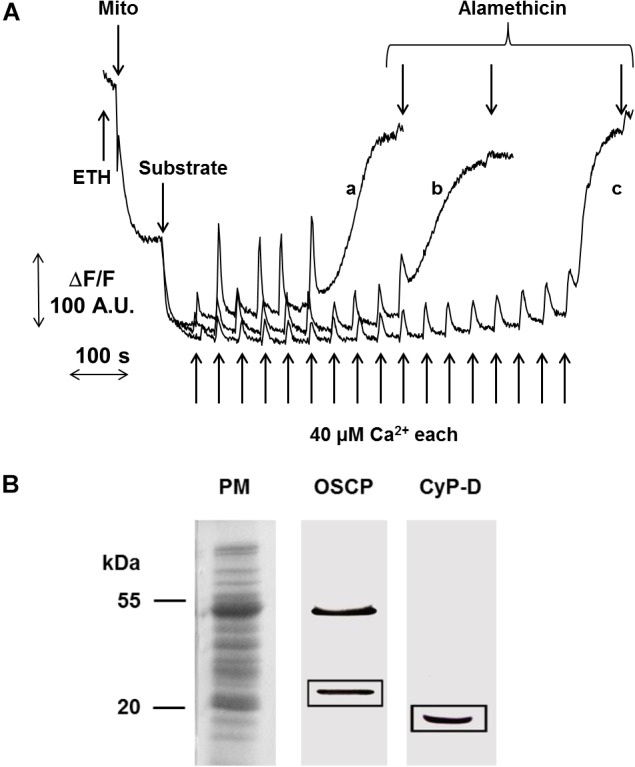
Effect of different substrates on PTP opening and detection of CyPD. **(A)** ΔΨ was monitored as fluorescence decrease of safranine O, the incubation medium was as in Figure 1A. CM were energized by the addition of either 5 mM succinate-Tris (trace a), or 5 mM succinate-Tris plus 1 mM pyruvate (trace b), or 10 mM malate plus 10 mM glutamate (trace c). PTP opening was induced by pulses of 40 μM Ca^2+^. **(B)** PM were subjected to SDS-PAGE and electroblotted onto nitrocellulose membrane. OSCP and CyPD were detected by the respective specific antibodies.

In pea stem crude mitochondria (CM), spontaneous Ca^2+^ transport into the matrix of energized mitochondria was not detectable (Figure 1A, trace a). Therefore, the presence of the Ca^2+^ ionophore ETH129 became necessary for matrix Ca^2+^ loading to trigger the PT as detected by ΔΨ (Figure 1A, traces b and c) and CRC (Figure 1B) measurements. CM were added to a medium containing safranine O and energized by 5 mM succinate. Pulses of 80 μM Ca^2+^ were added at regular time intervals. After approximatively four consecutive pulses, mitochondria underwent a sudden and spontaneous ΔΨ collapse, and the new steady state was unaffected by the final addition of the unselective pore-forming compound alamethicin (Figure 1A, trace b). PT occurrence was delayed in the presence of 0.5 μM CsA, and about twice as much Ca^2+^ was required to induce ΔΨ collapse (Figure 1A, trace c). These results were confirmed by CRC experiments (Figure 1B), where ETH129-mediated Ca^2+^ uptake by mitochondria was followed by Ca^2+^ release after four additions of 80 μM Ca^2+^ (Figure 1B, trace a). Consistent with a role of the PTP in Ca^2+^ release, the process was delayed by the addition of 0.5 μM CsA (Figure 1B, trace b). Mitochondrial respiration was maintained after ΔΨ collapse and Ca^2+^ release (Supplementary Figure S1) indicating that, even when ΔΨ was collapsed and followed by Ca^2+^ release, the mitochondrial electron transport chain was still active. In order to minimize the chances of probe artifacts, the experiments were also performed with rhodamine 123 (Supplementary Figure S2). The experimental pattern was very similar to that obtained with safranine O, but with a higher background noise. We next investigated the effect of respiratory substrates, which are known to affect the PTP of mammals (Fontaine et al., 1998). Compared with succinate alone (Figure 2A, trace a), higher Ca^2+^ loads were needed to induce PT opening with succinate plus pyruvate (Figure 2A, trace b), or with malate plus glutamate (Figure 2A, trace c). Interestingly, rotenone did not influence the PT (Supplementary Figure S3A), a finding which is in agreement with the presence of rotenone-insensitive NAD(P)H dehydrogenases in the inner membrane of plant mitochondria (Melo et al., 1996). Consistent with the inhibitory effects of CsA, Percoll-purified mitochondria (PM) contained a band with an apparent MW of 18 kDa that was recognized by the antibody against CyPD. Two bands were identified by the antibody against the mammalian OSCP subunit of F-ATP synthase, which showed an apparent MW of about 23 and 46 kDa, respectively (Figure 2B).

**FIGURE 3:**
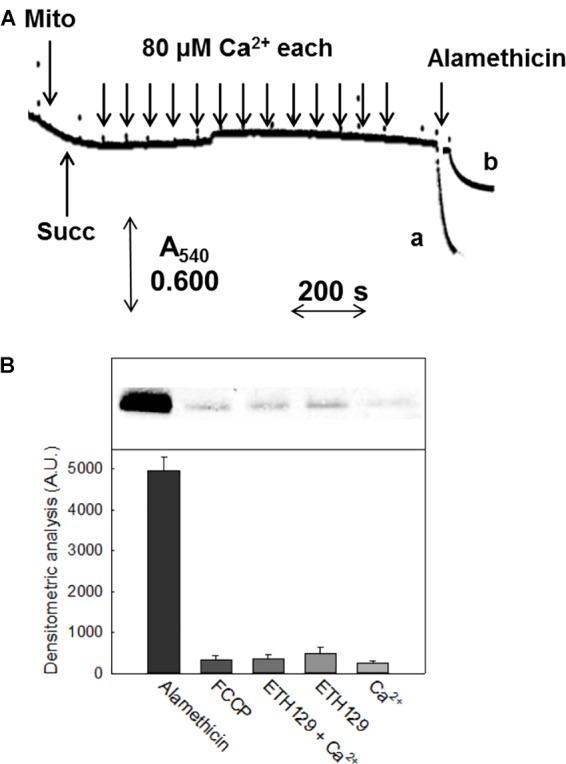
Swelling and release of Cyt *c* in CM. **(A)** Experimental conditions were as indicated in Figure 1. CM were incubated in 0.25 M non-ionic osmoticum (sucrose or mannitol) (trace a) or 0.125 M ionic osmoticum (NaCl, KCl, choline-Cl) (trace b). Swelling was monitored as absorbance decrease at 540 nm. **(B)** Immunoblot assay of released proteins from isolated mitochondria incubated in sucrose-based medium in the presence of different reagents, after SDS-PAGE. Mitochondrial protein (1 mg) was incubated in the assay buffer with 5 mM succinate, 1 mM Pi-Tris, 10 μM EGTA-Tris. From the left, lanes correspond to: addition of 5 μM alamethicin; 5 μM FCCP; 300 μM Ca^2+^ plus 10 μM ETH129; 10 μM ETH129; 300 μM Ca^2+^.

In mammals and *S. cerevisiae*, but not *D. melanogaster* (von Stockum et al., 2011), the PT is followed by swelling in sucrose-based media. In pea stem mitochondria onset of the PT was not accompanied by matrix swelling (Figure 3A), which was instead observed after the addition of 5 μM alamethicin both in sucrose (trace a) and in K^+^-containing media (trace b). Consistently, immunochemical experiments showed that Cyt *c* was not released from mitochondria unless alamethicin was added (Figure 3B).

**FIGURE 4:**
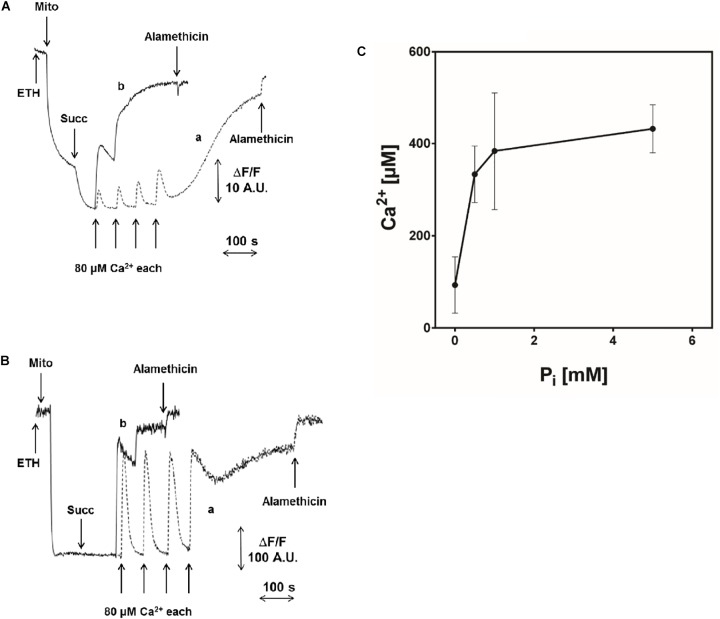
Effect of Pi on PTP opening induced by Ca^2+^. Experimental conditions were as indicated in Figure 1. **(A)** ΔΨ was monitored with 5 μM safranine O as fluorescence decrease; **(B)** CRC was evaluated with 0.5 μM calcium green-5N as fluorescence increase. Where indicated, 5 mM succinate-Tris (Succ) and 80 μM Ca^2+^ pulses in sequence were added. In traces a (controls), 1 mM Pi-Tris was added; in traces b, no Pi-Tris was present. **(C)** Isolated CM were incubated as in Figure 1, and Ca^2+^ was added to induce PTP opening, measured as fluorescence change of 0.5 μM calcium green-5N, in the presence of increasing concentrations of Pi-Tris.

Another interesting feature of pea stem PT was the inhibitory effect of increasing concentrations of Pi (Figures 4A–C), which is similar to what has been reported in Drosophila (von Stockum et al., 2011) and yeast (Jung et al., 1997; Cabrera-Orefice et al., 2010; Carraro et al., 2014) mitochondria, while Pi has a PT-inducing effect in mammals. In order to rule out that the observed inhibition was due to alterations of Δp components caused by Pi uptake (decreased ΔpH and increased ΔΨ) (Bernardi and Pietrobon, 1982), a similar experiment was performed in the presence of 10 mM Tris-acetate (pH 7.5), which causes similar changes in the proton-motive force. Under these conditions, PT opening was not significantly affected by acetate and occurred after the same number of Ca^2+^ pulses as in the control (i.e., in absence of Pi) (Supplementary Figure S3B).

The effect of other well-characterized modulators of PT was assessed next (Table 1). PT was stimulated by thiol oxidants (diamide and phenylarsine oxide, PheAsO), Bz-423 and oligomycin, inhibited by Mg-ADP and Mg-ATP. The most unexpected finding, however, was the PTP-inducing effect of oligomycin.

**Table 1:** Effect of permeability transition pore (PTP) modulators on CRC in CM.

	Control Ca^2+^ (μM)	Treatment Ca^2+^ (μM)	% of control
Diamide (2 mM)	369 ± 95	278 ± 80^**^	75
PheAsO (50 μM)	353 ± 91	188 ± 61^**^	53
Bz-423 (100 μM)	384 ± 159	199 ± 66^*^	52
Oligomycin (1 μM)	380 ± 120	122 ± 24^**^	32
Mg-ADP (2 mM)	412 ± 296	701 ± 258^*^	170
Mg-ATP (2 mM)	260 ± 83	480 ± 126^**^	184

*Isolated CM were incubated in 0.25 M sucrose, 10 mM MOPS-Tris (pH 7.4), 1 mM P_i_-Tris, 10 μM EGTA-Tris, 10 μM ETH129 and in the presence of different PTP modulators. Data are means ± SD of at least five independent replicates. Each treatment was compared with its relative control and analyzed by paired sample t-test (^∗^p < 0.05, ^∗∗^p < 0.01)*.

### Characterization of Pea Stem F-ATP Synthase

**FIGURE 5:**
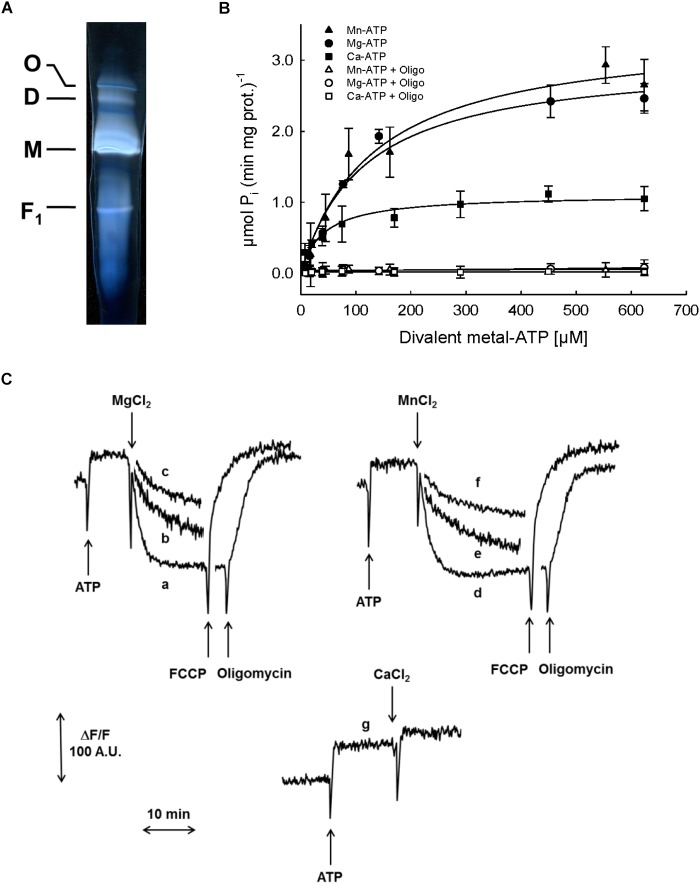
Characterization of F-ATP synthase. **(A)** Detection of F-ATP synthase complexes. PM were solubilized by digitonin and separated by BN-PAGE. The active bands, corresponding to F-ATP synthase oligomer (O), dimer (D), monomer (M) and F_1_ were visualized by zymogram as white bands on dark background. **(B)** Evaluation of F-ATP synthase activity in SMP as ATP hydrolysis, in the presence of increasing concentrations of each divalent metal (Mn^2+^, closed triangles; Mg^2+^, closed circles; Ca^2+^, closed squares) with ATP, and 1 μM oligomycin (Mn^2+^, open triangles; Mg^2+^, open circles; Ca^2+^, open squares). **(C)** Evaluation of F-ATP synthase proton pumping activity in SMP as fluorescence quenching of AO induced by ATP and divalent metals (Mn^2+^, Mg^2+^, or Ca^2+^). Traces a: 800 μM Mg-ATP; b: 400 μM Mg-ATP; c: 200 μM Mg-ATP; d: 800 μM Mn-ATP; e: 400 μM Mn-ATP; f: 200 μM Mn-ATP; g: 800 μM Ca-ATP. The proton gradients were collapsed by either 5 μM FCCP or 1 μM oligomycin.

Since the F-ATP synthase of mammals, *S. cerevisiae* and *D. melanogaster* can be turned into Ca^2+^-dependent channel corresponding to PTP (Bernardi et al., 2015), we studied this enzyme in pea stem mitochondria. Blue native PAGE of PM after treatment with digitonin revealed the presence of monomers, dimers and oligomers with clear in-gel ATPase activity (Figure 5A). The hydrolytic activity in SMP, evaluated as Pi release (Figure 5B), was observed in the presence of all the divalent cations tested. However, the concentration-dependence of hydrolysis rate with Mg-ATP and Mn-ATP showed similar affinity for the enzyme (*V*_max_ = 3.0 and 3.4 μmol (min mg prot)^-1^, respectively; *K*_m_ = 115 and 128 μM, respectively), whereas both kinetic parameters for Ca-ATP were lower [*V*_max_ = 1.1 μmol (min mg prot)^-1^, and *K*_m_ = 40 μM], indicating that all these cations support ATPase activity with similar efficiency. In all these experiments, oligomycin was able to completely inhibit ATP hydrolysis.

The proton pumping activity of F-ATPase was further characterized in SMP based on the fluorescence quenching of AO, which takes place when the vesicle lumen undergoes acidification (Figure 5C). In the presence of either Mg^2+^ (traces a, b and c) or Mn^2+^ (traces d, e and f), ATP induced the formation of a proton gradient, evaluated as decrease in fluorescence of AO, until a steady-state was reached. Subsequent addition of either oligomycin or the protonophore agent FCCP induced complete recovery of the fluorescence, indicating that the transmembrane proton gradient established by ATP hydrolysis had been completely dissipated. Remarkably, and in spite of the Ca-ATPase activity that we just documented, no proton gradient was formed across the SMP membrane when Ca^2+^ was added in the presence of ATP (trace g).

## Discussion

In this work we have defined the essential features of the PT in pea stem mitochondria. We found that such mitochondria exhibited PT features resembling those already described for potato, mammals, yeast, and Drosophila mitochondria (Arpagaus et al., 2002; Cabrera-Orefice et al., 2010; Giorgio et al., 2013; Carraro et al., 2014; von Stockum et al., 2015). PT opening in pea was induced by Ca^2+^ and stimulated [“sensitized,” (Bernardi et al., 2015)] by diamide, PheAsO and Bz-423 (Table 1) and, conversely, the onset of permeabilization could be delayed (“desensitized”) by CsA and F-ATP synthase substrates, such as Mg-ADP and Mg-ATP. PT was induced by Ca^2+^ loading (corresponding to a total added concentration between 240 and 720 μM) only in the presence of the Ca^2+^ ionophore ETH129, in contrast to what was reported in mitochondria isolated from potato. The latter showed a spontaneous Ca^2+^ uptake causing PT and mitochondrial swelling that is inhibited by CsA in the presence of antioxidants (Arpagaus et al., 2002). Our results are similar to what was found in *S. cerevisiae*, where mitochondria lack a Ca^2+^ uniporter and PT occurs only after the addition of ETH129 (Yamada et al., 2009; Carraro et al., 2014), unlike in *Yarrowia lipolytica* and *Endomyces magnusii*, which are strikingly resistant to induction of the permeability transition by Ca^2+^ (Kovaleva et al., 2009). Therefore, the only common feature required for PT manifestation in the species so far examined appears to be the dependence on matrix Ca^2+^ (Table 2). Furthermore, PTP open probability seems to be dependent on the substrate used to induce Δ*p* formation, since the Ca^2+^ concentration required for PTP activation is lower with succinate than with malate and glutamate. This is in apparent contrast with what has been observed in mitochondria from skeletal muscle where PTP opening required more Ca^2+^ with succinate in the presence of rotenone (Fontaine et al., 1998), which was later shown to be an inhibitor of the PTP, possibly due to lower ROS production (Chauvin et al., 2001). Our results are in agreement with what has been described for plant mitochondria, which possess alternative electron pathways and succinate oxidation is the main process leading to ROS production (Braidot et al., 1999; Casolo et al., 2000; Jardim-Messeder et al., 2015).

**Table 2:** Comparison among some features of mitochondrial PT in different species.

	*Mammals*	*Drosophila melanogaster*	*Saccharomyces cerevisiae*	*Pisum sativum*
Ca^2+^ induction	Yes^1^	Yes^2^	Yes^3^ With ETH129^4,5^	With ETH129^17^
Inhibition by CsA	Yes^6-9^	No^2^	Yes^3^/No^4-6^	Yes^17^
Effect of Pi	Activation^10^	Inhibition^2^	Inhibition^4-6^	Inhibition^17^
Swelling	Yes^11,12^	No^2^	Yes^3-6^	No^17^
Effect of Oligomycin	Inhibition^13,14^ Activation^15,16^	n.d.	No effect^4^	Activation^17^

*^1^Haworth and Hunter (1979); ^2^von Stockum et al. (2011); ^3^Kamei et al. (2018); ^4^Jung et al. (1997); ^5^Yamada et al. (2009); ^6^Carraro et al. (2014); ^7^Fournier et al. (1987); ^8^Crompton et al. (1988); ^9^Broekemeier et al. (1989); ^10^Gunter and Pfeiffer (1990); ^11^Davidson and Halestrap (1990); ^12^Hunter et al. (1976); ^13^Novgorodov et al. (1990); ^14^Shchepina et al. (2002); ^15^Sokolova et al. (2013); ^16^Peachman et al. (2001); ^17^present work; n.d., not determined.*

In plants, Ca^2+^ transport into the mitochondrial matrix could be mediated by a Pi symporter (Dieter and Marmé, 1980; Åkerman and Moore, 1983; Silva et al., 1992) or a Ca^2+^ uniporter (Zottini and Zannoni, 1993). In addition, the homolog of the mammalian mitochondrial Ca^2+^ uniporter (MCU) described in plants (Bick et al., 2012; Stael et al., 2012; Rikhvanov et al., 2014) is strictly regulated by MICU (Wagner et al., 2015). Therefore, it will be interesting to understand why pea stem mitochondria do not show appreciable Ca^2+^ uptake and, hopefully, find the conditions to induce such a transport. We speculate that when oxygen is a limiting factor in plant organs (e.g., potato tuber) or during environmental stresses (e.g., waterlogging), it might favor the sensitized state of mitochondria and the Ca^2+^ loading as inducing factor.

In pea stem mitochondria the PT was modulated by CsA, which specifically interacts with mitochondrial CyPD. This protein has been described in Arabidopsis, where two single-domain mitochondrial cyclophilins, AtCYP21-3 and AtCYP21-4, are present (Romano et al., 2004). Unfortunately, no cyclophilins from *P. sativum* are present in protein databases, but in soybean (*Glycine max*, belonging to Fabaceae family like *Pisum*), among 62 putative cyclophilins, four are targeted to mitochondria (GmCYP49, GmCYP51, GmCYP61, and GmCYP62) and, possibly, also a fifth (GmCYP31) (Mainali et al., 2014). In particular, GmCYP49, 51, 61, and 62 share a high similarity and are therefore good candidates for the equivalent of CyPD in soybean.

It has been recently shown that mammalian OSCP binds CyPD by electrostatic interactions at helices 3 and 4, where also Bz-423 binds (Stelzer et al., 2010; Giorgio et al., 2013). In the protein database for *Pisum*, a short fragment of OSCP sequence is present (38 aa, accession n. P17604) which shows 81% identity with soybean OSCP (accession n. XP_003546884). Comparison of the latter with bovine OSCP (accession no. P13621) shows that the amino acids present in the binding site for CyPD (E17, D94, E98, and F101) are well conserved in soybean (N96, D121, E125, and F128), suggesting that these residues could be involved in CyP binding in plants.

Another peculiar feature of pea stem mitochondria is related to the effect of oligomycin (the selective inhibitor of F-ATP synthase), which sensitized the PTP to Ca^2+^-dependent opening, while in mammals both inhibition and stimulation have been reported (Novgorodov et al., 1990; Peachman et al., 2001; Shchepina et al., 2002; Sokolova et al., 2013). Interestingly, Ca-ATPase activity of F-ATP synthase of CM showed full sensitivity to oligomycin, indicating that catalysis should be coupled to proton translocation, which occurs through the two aqueous half transmembrane channels formed by the a subunit and the c-ring (Guo et al., 2017). On the other hand, Ca-ATPase activity was unable to generate (or maintain) a proton gradient across the inner mitochondrial membrane, suggesting the possible occurrence of H^+^ backflow, which would be consistent with PTP opening. Our finding is fully consistent with a similar observation in SMP from beef heart (Papageorgiou et al., 1998) and supports the hypothesis that also in pea stem mitochondria the PTP may originate from a Ca^2+^-dependent conformational transition of F-ATP synthase, an issue that is matter of controversy. This theory is supported by studies where F-ATP synthase had been genetically manipulated (Bonora et al., 2013; Giorgio et al., 2013), by measurements of channel activity by electrophysiology (Giorgio et al., 2013; Alavian et al., 2014; Carraro et al., 2014; von Stockum et al., 2015) and by F-ATP synthase mutagenesis at specific residues (Giorgio et al., 2017; Antoniel et al., 2018; Guo et al., 2018). Conversely, it has been questioned by studies where subunit c (He et al., 2017b) or peripheral subunits b and OSCP (He et al., 2017a) were genetically ablated in HAP1 cells. These cells still underwent a CsA-sensitive PT. However, and as noted elsewhere (Bernardi and Lippe, 2018), PTP-dependent swelling rates in KCl were drastically reduced in mitochondria lacking peripheral stalk subunits, suggesting that the pore size was actually affected by the deletion, as also indicated by a recent study (Carraro et al., 2018).

We have tried to tackle this question more directly by eluting the enzyme from native gels such as the one depicted in Figure 5A, followed by incorporation of the eluted proteins in lipid bilayers as reported previously (Giorgio et al., 2013). Current elicited by Ca^2+^ and Bz-423 could be detected, but not univocally assigned to the F-ATP synthase because they could not be consistently blocked by PTP inhibitors (results not shown), a possible consequence of contamination by VDAC, which gives rise to currents similar to PTP (Szabó and Zoratti, 1993; Szabó et al., 1993). We are also testing whether the reconstitution experiments can be improved by the addition of cardiolipin, which has been shown to play a role in PTP modulation (Zhou et al., 2017).

It has recently been proposed that the AAA-protease SPG7 (paraplegin, the product of the SPG7 gene, Casari et al., 1998) may be involved in the formation of the PTP (Shanmughapriya et al., 2015). As noted elsewhere (Bernardi and Forte, 2015), no direct evidence has been provided that SPG7 can actually participate in formation of a channel, and the conclusions have been challenged by two independent studies that attribute the effects to defective processing of components of the MCU complex, which would indirectly affect the PTP (König et al., 2016; Tsai et al., 2017). Given that in pea stem mitochondria the MCU was not active in Ca^2+^ uptake, we think that SPG7 is unlikely to play a role in the PT described here.

Pi is the most puzzling amongst PTP effectors. In beef heart mitochondria, CyPD binding to OSCP is favored by Pi (Giorgio et al., 2009) and this interaction lowers the concentration of Ca^2+^ needed to open the PTP (Giorgio et al., 2013). In keeping with this, PTP of CyPD-null mouse liver mitochondria is inhibited by matrix Pi (Basso et al., 2008). Even if pea stem mitochondria possessed a mitochondrial CyP and PTP was modulated by CsA, in this species Pi acted as an inhibitor of PT. This effect seems not to be dependent on a perturbation of the Δ*p*, since PTP opening was not significantly altered when acetate replaced Pi. This behavior is similar to what found in *S. cerevisiae*, *Debaryomyces Hansenii*, and *D. melanogaster* (Jung et al., 1997; Yamada et al., 2009; Cabrera-Orefice et al., 2010; von Stockum et al., 2011; Carraro et al., 2014). Nevertheless, Drosophila appears not to contain a mitochondrial cyclophilin and the mitochondrial CyP isoform in *S. cerevisiae* appears not to regulate the PTP, consistently with the insensitivity of the PTP of both organisms to CsA (Bernardi et al., 2015). It should be mentioned that agar-embedded yeast mitochondria display a CsA-sensitive PT, adding a further layer of complexity to the known factors of PTP modulation (Kamei et al., 2018). We suggest that in pea mitochondria increasing Pi concentrations lead to decreased matrix free Ca^2+^ (Zoccarato and Nicholls, 1982), which prevails on any PTP-promoting effect of Pi. Further experiments are needed to clarify whether a PTP-regulatory interaction of a matrix CyP with F-ATP synthase takes place in plant mitochondria, since such a protein, even if present in PM, was not detected in purified F-ATP synthase dimers after BN-PAGE (result not shown). This could depend on the presence of digitonin or on the buffer ionic strength, both necessary to isolate functional dimers by BN-PAGE. These experimental conditions could have induced the detachment of CyP from the enzymatic complex, which was proven to be associated by electrostatic interactions (Giorgio et al., 2013).

A common feature of the PT of pea stem mitochondria and the Ca^2+^-induced Ca^2+^-release channel of Drosophila is the lack of swelling, which is consistent with the absence of Cyt *c* release from mitochondria and suggests a low conductance of the PTP in these species. This mechanism for Ca^2+^ release might act in synergy with K^+^ channels, which are involved in the modulation of mitochondrial permeability during responses to environmental/oxidative stress and in the control of ROS production in plants (Vianello et al., 2012; Trono et al., 2015).

The emerging view of the structure and function of PT/PTP has many puzzling aspects. The PTP appears to be formed by a protein that has not primarily evolved and selected to perform this function. Rather, it appears to be constituted by more than one protein, its activity being modulated in distinct ways in different species. Recent evidence suggests that PTP may be constituted by F-ATP synthase and possibly other proteins with regulatory functions (Bernardi et al., 2015). This is an intriguing observation, because F-ATP synthase is one of the essential 1500 “nanomachines” that have been suggested to have been essential for life to emerge and for survival during evolution (Falkowski, 2015).

It has been previously suggested that PT/PTP may have co-evolved in early eukaryotes with other mitochondrial metabolite transport systems to regulate the endosymbiont-host relationships (Vianello et al., 2012), following the establishment of endosymbiosis (Margulis, 1975) between an aerobic α-proteobacterium and a proto-eukaryote or, alternatively, a member of the Rickettsiales (*Midichloria mitochondrii*) and an archaebacterium, 1.5–1.8 billion years ago (Sassera et al., 2011). This hypothesis is corroborated by the observation that the F-ATP synthase in prokaryotes does not assemble into dimers (Dabbeni-Sala et al., 2012), a conformation that was therefore acquired only after the endosymbiotic event. Dimer formation seems to be dependent on several membrane-bound subunits (Guo et al., 2017), which contribute to stabilize the monomer interactions and, at the same time, to bend the inner mitochondrial membrane (Paumard et al., 2002). In this new conformation, F-ATP synthase might confer novel properties, including the ability to form a pore. Therefore, the emergence of PT/PTP may not be simply explained by a Darwinian mechanism, based on a mutation followed by natural selection (adaptation). Rather, within the framework of the so-called “extended synthesis” (Vianello and Passamonti, 2016), it may be explained by a mechanism of pre-adaptation (*sensu* Darwin), defined cooptation as exaptation by Gould and Vrba (1982).

In the light of the above explanation, the differences in structure and regulation shown by PTP in phylogenetic distant species suggest that the function of PTP could be manifested in parallel in different eukaryotic lineages, in a form that could be defined convergence at the molecular level.

## Author Contributions

EP, VCa, EB, GL, AV, VG, MZ, and PB designed the study. VDC, EP, VCa, EB, GL, AF, CP, SP, AB, VG, VCh, and MZ performed parts of the lab work and analyzed and interpreted the data. VDC, EP, VCa, EB, GL, VG, AV, MZ, and PB contributed to the interpretation of the results and valuable discussion. VDC, EP, VCa, EB, GL, VG, AV, PB, and MZ contributed to the drafting of the work. All authors finally approved the version to be published and agreed to be accountable for all aspects of the work in ensuring that questions related to the accuracy or integrity of any part of the work are appropriately investigated and resolved.

## Conflict of Interest Statement

The authors declare that the research was conducted in the absence of any commercial or financial relationships that could be construed as a potential conflict of interest.
