# Supplementary material for: Properties of the Permeability Transition of Pea Stem Mitochondria
Source: Front Physiol. 2018 Nov 21;9:1626. doi: 10.3389/fphys.2018.01626 (PMC6262314; doi:10.3389/fphys.2018.01626)
Supplement: Supplementary file 1 [file Data_Sheet_1.docx]

**SUPPLEMENTARY MATERIAL**

**FIGURE 1 Suppl. |** Detection of oxygen consumption in CM. Isolated CM were incubated in 0.25 M sucrose, 10 mM MOPS-Tris pH 7.4, 1 mM Pi-Tris, 10 µM EGTA-Tris, 10 µM ETH129. Where indicated, 5 mM succinate-Tris, pulses of 80 µM Ca^2+^ and 1 mM KCN were added.

**FIGURE 2 Suppl. |** PTP opening evaluated as ΔΨ collapse with Rhodamine 123 in CM. Isolated CM were incubated in 0.25 M sucrose, 10 mM MOPS-Tris pH 7.4, 1 mM Pi-Tris, 10 µM EGTA-Tris, 10 µM ETH129. The incubation medium was supplemented with 0.5 µM Rhodamine 123 and ΔΨ collapse was monitored as fluorescence decrease. Where indicated, 5 mM succinate-Tris and pulses of 40 µM Ca^2+^ were added.

**FIGURE 3 Suppl. |** Effect of rotenone and acetate on PTP opening in CM. Isolated CM were incubated in 0.25 M sucrose, 10 mM MOPS-Tris (pH 7.4), 10 µM EGTA-Tris and 10 µM ETH129, with (traces a and b) or without (traces c and d) 1 mM Pi-Tris. The incubation medium was supplemented with 5 µM safranine O and ΔΨ was monitored as fluorescence decrease; trace a, control (1 mM Pi); trace b, 2 µM rotenone was added to the incubation medium; trace c, control (no Pi); trace d, 10 mM acetate-Tris was added to the incubation medium.

**Figure 1 Suppl.**

**
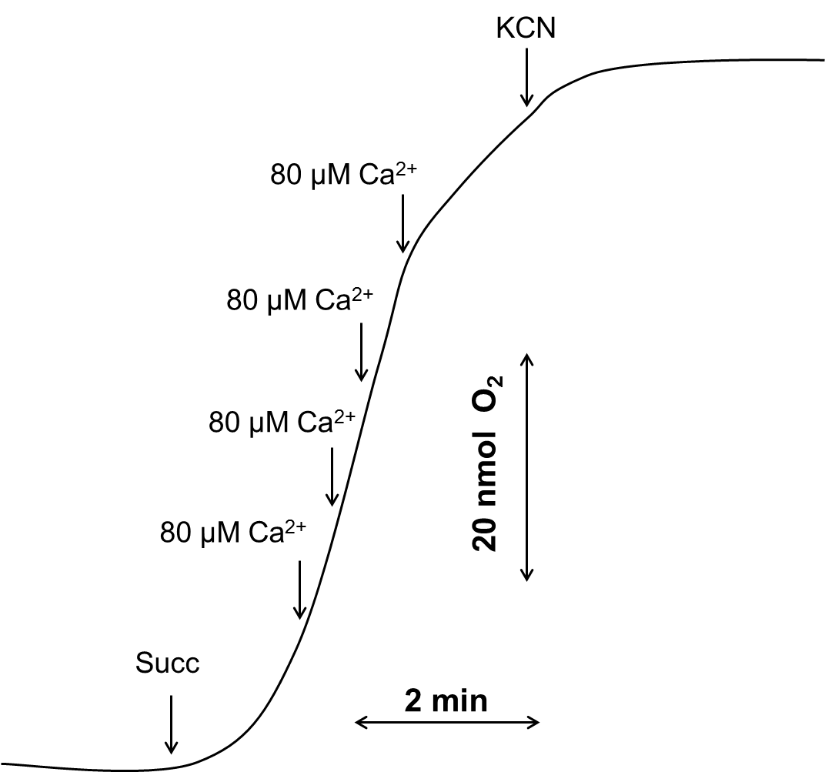
**

**Figure 2 Suppl.**


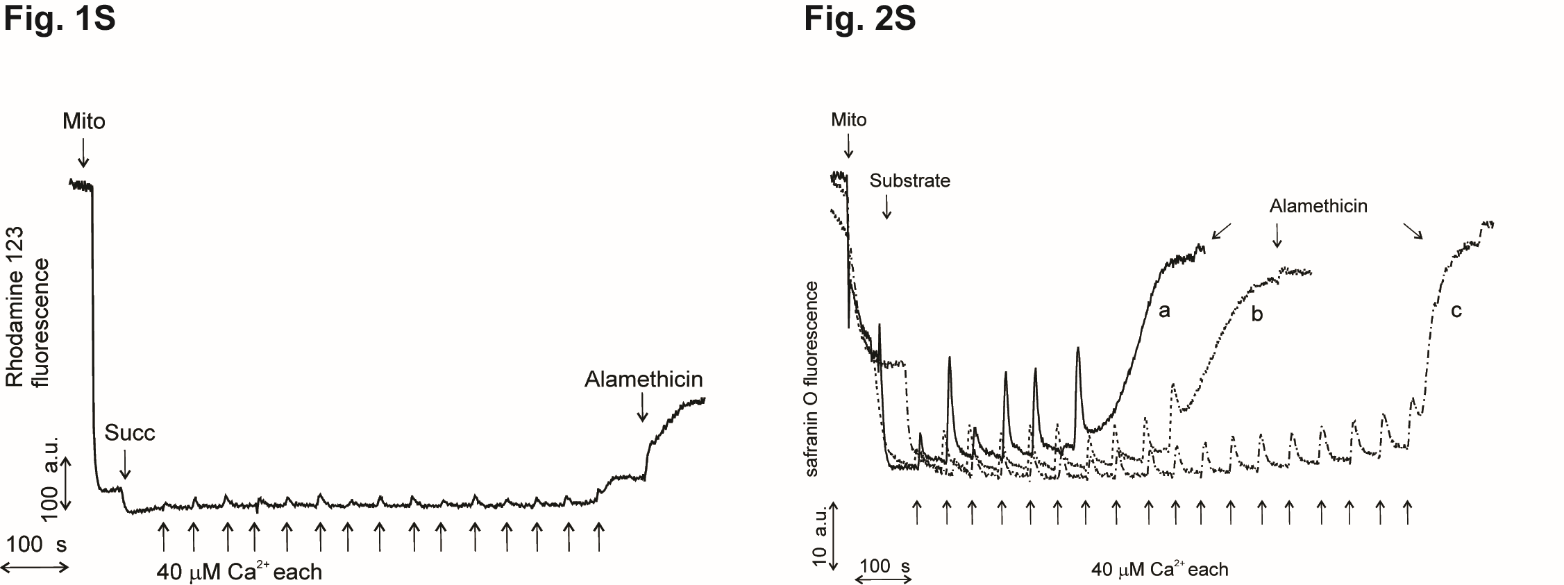


**Figure 3 Suppl.**

**
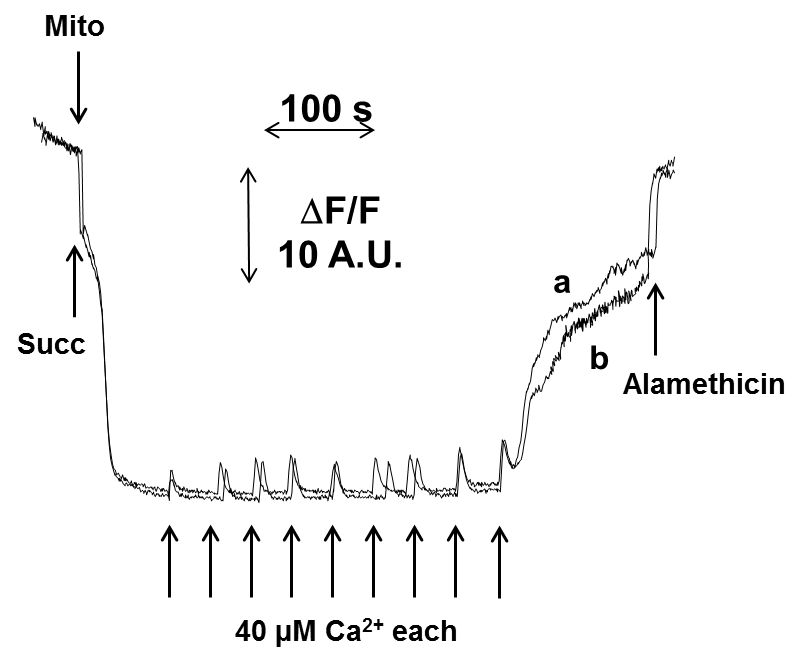
 A**

**
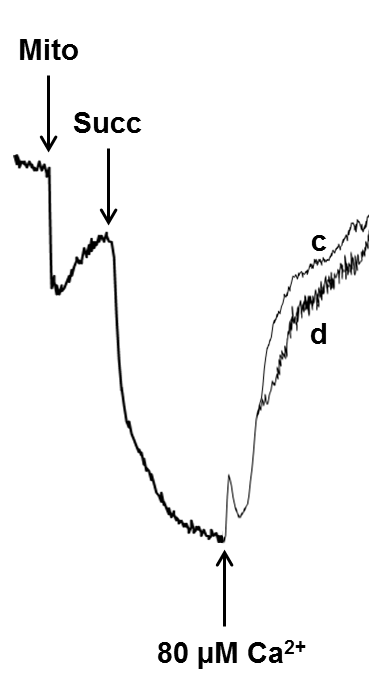
**

**B**
